# Social and structural determinants of emergency department use among Arab and Jewish patients in Jerusalem

**DOI:** 10.1186/s12939-022-01698-1

**Published:** 2022-11-07

**Authors:** Shifra Unger, Zvika Orr, Evan Avraham Alpert, Nadav Davidovitch, Ilana Shoham-Vardi

**Affiliations:** 1 Faculty of Life and Health Sciences, Jerusalem College of Technology, Jerusalem, Israel; 2 Department of Emergency Medicine, Shaare Zedek Medical Center, Faculty of Medicine, The Hebrew University of Jerusalem, Jerusalem, Israel; 3 Faculty of Health Sciences, Department of Public Health, Ben-Gurion University of the Negev, Beersheba, Israel

**Keywords:** Emergency Department, Repeat Visits, Structural Competency, Structural Vulnerability, Social Determinants of Health, Inequity, Israel

## Abstract

**Background:**

A growing body of research demonstrates that economic conditions and racial and ethnic disparities result in excessive overuse of emergency departments (EDs) by a small group of socioeconomically marginalized residents. Knowledge and understanding of these issues on the part of the healthcare team can promote equality by providing structurally competent care. This study aims to identify the major social and structural factors related to patterns of ED visits by Arab and Jewish patients in Israel, where access to health services is covered by universal national health insurance.

**Methods:**

A cross-sectional study was conducted using questionnaires of ED patients in a tertiary care medical center in Jerusalem. The hospital is the largest of the three EDs in Jerusalem with over 90,000 adult patient visits a year. The sample was stratified by ethnicity, including 257 Jewish patients and 170 Arab patients. The outcome variable was repeat visits for the same reason to the ED within 30 days.

**Results:**

There were differences between Jewish and Arab patients’ social and structural characteristics, including health status, socioeconomic status, feeling of safety, and social support. There were also significant differences in some of the characteristics of health service utilization patterns, including ED repeat visits, language barriers when seeking healthcare in the community, and seeking information about medical rights. The variables associated with repeat visits were different between the two groups: among the Arab patients, repeat visits to the ED were associated with concerns about personal safety, whereas among the Jewish patients, they were associated with poverty.

**Conclusion:**

The study illustrates the gaps that exist between the Arab and Jewish population in Israel. The findings demonstrated significant differences between populations in both health status and access to health services. In addition, an association was found in each ethnic group between different structural factors and repeat ED requests. This study supports previous theories and findings of the relationship between structural and social factors and patterns of health services utilization.

**Supplementary Information:**

The online version contains supplementary material available at 10.1186/s12939-022-01698-1.

## Background

The Emergency Department (ED) is an important interface between the hospital and the community. As the ED serves all residents with medical needs, the patient population reflects the religious, linguistic, cultural, and economic diversity of the neighborhood and city [1]. Social scientists have studied how the diversity of one’s background impacts their help-seeking behavior in the ED [2].

Gaps and disparities in health exist in Israel similar to anywhere in the world. Braveman defined health disparity (which is the same as health inequality) as “a particular type of difference in health or in the most important influences on health that could potentially be shaped by policies. It is a difference in which disadvantaged social groups (such as the poor, racial/ethnic minorities, women, or other groups that have persistently experienced social disadvantage or discrimination) systematically experience worse health or greater health risks than more advantaged groups” [3]. In Israel, where access to healthcare is covered by national insurance, the gaps and inequalities in health status and indicators are rooted in class, ethnicity, nationality, religion, education, profession, place of residence, as well as in income [4]. For example, people from low-income households are 3.6 times more likely to have diabetes [5]. Also, there are differences in health status, morbidity, and mortality between Arabs and Jews in Israel [6–8], when mortality differentials for people older than 45 that emerged over the years have gradually widened [9]. In addition, there are also differences in access to health services [10].

Inequalities are reflected in social factors (race, ethnicity, socioeconomic status, civil status, language, education, place of residence, location of birth and emigration, gender, and age). These factors are affected by determinants that stem to a large extent, from structures such as economic systems, health policies, laws, and regulations. Examples of these determinants include the availability of resources to meet daily needs; discrimination; exposure to crime; public safety; and residential segregation [11].

While structural inequalities are recognized by public health professionals, it is important that those who care for patients at the bedside also be aware of these gaps. Metzl and Hansen suggested that the acquisition of such awareness and skills among healthcare providers should be referred to as *structural competency* [12]*.*

Structural competency is the trained ability to discern and acknowledge how sociopolitical structures, such as public policies, economic systems, and healthcare delivery organizations, have produced and maintained social inequities and health disparities [12]. These social inequities are often based on issues of race, ethnicity, religion, class, citizenship status, language, geography, gender, and age. Structural competency calls on healthcare professionals to recognize how these structures shape diseases and symptoms. It also encourages them to be aware of how these inequalities manifest themselves in provider patient interactions, as well as at the community and policy levels [12].

A growing body of research in the United States demonstrates that economic conditions and racial and ethnic disparities result in the excessive “overuse” of EDs by a small group of socioeconomically marginalized residents [13, 14]. These repeat patients—often referred to as “frequent flyers”—keep returning to the ED because the underlying cause of their problem is not being treated [15]. Prescribing patients treatment recommendations that they cannot adhere to may increase repeat visits to the ED.

Accordingly, researchers suggested that vulnerable people (immigrants, the underinsured, the unemployed, etc.) are more likely to frequently visit EDs [16, 17]. Other factors associated with frequent ED use include physical and mental comorbidities [18] and the lack of community health services [19, 20].

As shown, health inequality does exist within and between populations throughout the world. Vulnerable populations are more likely to experience worse health outcomes and inappropriate health services usage patterns [21]. This study attempts to fill in the missing knowledge gap about structural inequalities in Israel and examine the association between repeated ED visits and structural factors among Arab and Jewish patients.

Israel is a multiethnic society inhabited by approximately 9 million people. Currently, the Israeli population includes 74.7% Jews, 20.8% Arabs, and 4.5% other ethnicities [22]. Most of the Arabs in Israel are Muslims (over 80%) and the remaining are Christians, Druze, Circassians, and other small minority groups. More than half of the Arab Israeli population live in the periphery (56%), especially in the northern periphery (42%), while another 33% reside in the major cities of Haifa and Jerusalem [23]. In Jerusalem, Arabs make up about 38% of the population; 19% of Arabs in Israel live in Jerusalem [22].

Arabs and Jews are highly segregated socially and residentially, living in separate communities. Even in the few cities that are considered mixed, the Arabs and the Jews do not usually share the same neighborhoods. Arabs in Israel are consistently lower in every aspect of stratification (i.e., politically, socially, and economically) than the Jewish majority, and in general they are located at the bottom of the Israeli ethnic stratification ladder [24].

In Israel, since 1994, according to the National Health Insurance Law, all legal residents (citizens as well as permanent residents) are entitled to health services based on the principles of justice, equality, and mutual assistance, provided through a legally defined basket of health services. The services are under the overarching responsibility of the state and are provided by the four nonprofit health maintenance organizations (HMOs). Premiums are graduated by income alone, irrespective of health status. Emergency medical services are provided unconditionally [25]. If, in retrospect, it turns out that the referral was not justified, the patient will pay for the treatment and the ambulance. While studies in the US have indicated a statistically significant correlation between socioeconomic status and use of the ED [26], it is not clear how this would apply to EDs in Israel, where all residents are covered under universal health insurance.

Jerusalem EDs have patients from diverse cultures, religions, and socioeconomic levels. In Jerusalem, general EDs operate in three hospitals. Also, there are Palestinian hospitals under the supervision of the Israeli Ministry of Health. The Israeli EDs serve the entire population, while Palestinian hospitals serve mainly Arabs.

## Methods

This is a cross-sectional study of ED patients at a tertiary care medical center in Jerusalem during June to October 2019. The hospital serves the diverse population of the city, including Christian and Muslim Arabs, and secular, traditional, national religious, and ultraorthodox Jews.

The data collection was conducted as part of a larger study that examined social and structural determinants of ED utilization and patient experience among Jewish and Arab patients in Jerusalem [27]. The study included ED patients over the age of 18. Interviewers who have been trained in research methods collected data by completing face-to-face questionnaires. The sample was stratified by ethnicity, to include a sufficient number of both Arabs (*n* = 170) and Jews (*n* = 257). The interviewers reached out to the patients in the ED, explaining the study, and only patients who signed written consent forms were interviewed. Data collection took place throughout the week in various shifts (morning, evening, night) to avoid selection bias. Ethical approval was obtained from the hospital Institutional Review Board known as the Helsinki Committee.

Following a pilot with 12 patients (both Arabs [*n* = 2] and Jews [*n* = 10]), the questionnaire was revised. The interview, using the final structured questionnaire, was conducted in Hebrew or Arabic by an interviewer fluent in one of the languages. The questionnaire was written in Hebrew and translated into Arabic by a professional translator. The translation was then compared to the original and edited by native Arabic speakers.

The study tool was a questionnaire with closed-ended questions. The questionnaire included measures of social and structural factors that are related to the use of the ED that were adapted from the literature [11, 15]. Social and structural factors included demographic characteristics (age, gender, marital status, number of children), sociodemographic background (socioeconomic status, education, employment, ethnic origin, religion, religiosity, civil status, language, use of welfare services, exposure to violence, sense of safety, social support), health status (general health status, chronic diseases, chronic pain), health services utilization (primary care physician [PCP] and ED visits) and access to health services (language barriers and difficulty with finding information about medical rights). Questions about current ED visit (mode of arrival, referral, escort, translation use, patient experience) were also included in the questionnaire. The questionnaire is enclosed in Additional file 1.

The questionnaire was constructed according to the conceptual framework of 'structural vulnerability' and includes social and structural factors that were found to be associated with ED visits in a study by Bourgois et al. [15]. The questions in the study were constructed according to the Structural Vulnerability Assessment Tool they suggested for medical staff. Some of the questions were taken from an existing valid and reliable questionnaire of the Israel Ministry of Health’ ED patient experience survey [28] including the items regarding health status and current ED visit. The questionnaire was validated by content validity and re-evaluated after the pilot. The internal reliability was also tested in the patient experience cluster of questions and found to be high (Cronbach's alpha stands at 0.856).

Repeat visit to the ED was defined as a self-reported repeat visit to the ED (to the same or a different hospital) for the same medical reason, within 30 days. The independent variables included: age, gender, marital status, language, ethnicity, health status, sense of safety, previous year primary care physician (PCP) visits, chronic diseases, chronic medications, chronic pain, education, employment, social support, use of welfare services, exposure to physical or emotional violence, illegal drug use, and alcohol use. Additional variables referred to characteristics of recurrent visits and the usage of health services in the community.

### Sample size

Sample size was calculated based on a similar study from the United States [29] using WinPepi software. US data were used as this is a preliminary study of this type in Israel and no previous Israeli data were available. The US study found that vulnerable groups tend to favor the ED as a first place of treatment (24% versus 13% in the comparison group; OR 2.24) [29]. The hypothesis was that the differences in repeat visits between Jewish and Arab patients would be similar. Accordingly and with an expected ratio of 1.5 between Jewish and Arab patients, to examine relationships between the variables described with a power of 80% and a 5% significance level, a sample of 338 patients was planned with 40% being Arabs and 60% Jews. In practice, slightly more were recruited and the number of participants in the study stands at 257 Jews and 170 Arabs.

### Statistical analysis

Data processing and analysis were done using IBM SPSS Statistics for Windows, Version 25.0 (Armonk, NY: IBM Corp). Statistical significance was determined as *p* < 0.05. Initially, descriptive statistics of the sociodemographic and health characteristics of the study participants were performed, followed by a univariate analysis of health services utilization patterns for the entire study population, and then stratified analysis for each of the research groups (Arabs and Jews). T-test, chi-squared, and Mann–Whitney tests were used according to the type of the variable. For the construction of the multivariable models, correlations between independent variables that were significantly associated with the outcome variable were examined. Multivariate models for each of the research groups were calculated using logistic regression. A model with the best fit was selected in reference to the significance of the model constant, Nagelkerke R-squared, likelihood ratio test, Hosmer–Lemeshow, and ROC analysis.

## Results

### Characteristics of study participants by ethnicity

Demographics and health characteristics are presented in Table 1.

**Table 1:** Demographics and health characteristics

**Characteristic**		Arabs (*n* = 170)	Jews (*n* = 257)	*p* value
Age, mean (SD)		50.7 (18.6)	52.1 (23.4)	unpaired t test t = 0.650 *p* = 0.516
Gender, %	Male	54.4	51.8	x^2^ = 0.587 *p* = 0.295
	Female	45.6	48.2	
Marital status, %	Single	13.0	22.7	x^2^ = 22.198 *p* < 0.001
	Married	79.3	58.2	
	Divorced / Separate	3.0	9.0	
	Widower	4.1	10.2	
Number of children (of those who have), Median (Interquartile Range [IQR])	25.6% have no children	5 (4–7)	4 (2–6)	U = 9010 *p* < 0.001
Familiar with welfare services, %		31.1	14.1	x^2^ = 17.712 *p* < 0.001
Physical violence^a^, %	Referral reason	1.2	0.4	x^2^ = 3.896 *p* = 0.048
	Always	0.0	0.0	
	Sometimes	1.8	0.4	
	Seldom	1.2	0.4	
	Never	95.8	98.8	
Mental violence^a^, %	Referral reason	1.2	0.0	x^2^ = 9.764 *p* = 0.002
	Always	1.2	0.0	
	Sometimes	6.6	2.0	
	Seldom	3.0	2.0	
	Never	88.0	96.0	
Sense of safety, %	Concern about safety	23.7	15.5	x^2^ = 4.454 *p* = 0.035
Social support, %	Always	74.0	85.3	U = 18,890 *p* = 0.004
	Usually	20.7	11.1	
	Sometimes	0.6	2.0	
	Never	4.7	1.6	
Substance use^a^, %	Always	0.0	0.0	x^2^ = 6.361 *p* = 0.012
	Sometimes	0.0	0.4	
	Seldom	0.6	0.8	
	Never	98.2	92.7	
	Have taken in the past	0.6	2.8	
	Medical cannabis	0.6	3.2	
Alcohol use^b^,%	Several times a day	0.0	0.4	
	Once a day	0.0	0.4	
	Several times a week	0.0	1.6	
	Never / less than once a week	100	97.6	
Socioeconomic status, %	Excellent	1.2	6.8	U = 13,998 *p* < 0.001
	Good	11.8	25.8	
	Fair	40.2	43.2	
	Not so good	32.0	14.8	
	Poor	14.8	9.3	
Education, %	No formal education	5.4	3.2	U = 11,267 *p* < 0.001
	Elementary school	38.7	3.6	
	Secondary school	31.5	32.5	
	Nonacademic higher education	6.5	11.9	
	Academic education	17.9	35.3	
	Religious seminary (Yeshiva)^c^	–	10.7	
Employment, %	Employed	46.8	38.1	x^2^ = 3.129 *p* = 0.077
Language, %	Does not speak Hebrew	47.3	3.5	x^2^ = 118.538 *P* < 0.001
Health status, %	Very good	33.1	52.3	U = 16,577 *p* < 0.001
	Fair/poor	35.5	31.3	
	Not very good	24.3	13.3	
	Poor	7.1	3.1	
Chronic disease, %	No chronic disease	34.7	54.4	x^2^ = 29.789 *p* < 0.001
	1 chronic illness	23.4	27.6	
	> 1 chronic illness	41.9	18.0	
Chronic pain, %	No pain	44.3	71.0	x = 32.601 *p* < 0.001
	Sometimes	13.8	9.0	
	More than a month	6.0	4.7	
	More than 6 months	35.9	15.3	
Citizenship status (residents)^d ^, %		66.9	0	
PCP visits last year, %	0	7.8	10.8	U = 16,013 *P* < 0.001 comparing ≤ 10 times vs. > 10 visits x^2^ = 25.276 *p* < 0.001
	1–5	38.9	54.8	
	6–10	16.2	18.8	
	More than 10	37.1	15.6	
Finding information about medical rights, %	With difficulty	4.7	7.1	x^2^ = 15.582 *p* < 0.001
	Without difficulty	27.2	44.3	
	Did not search	68.0	48.6	
Language barriers in community health care, %		26.0	3.5	x^2^ = 47.070 *p* < 0.001

^a^There were not enough cases to analyze. The comparison is between 'never' and all others

^b^not enough cases to analyze

^c^not relevant for Arabs

^d^Residency is the status of a person who lives permanently in the country but is not a citizen of it. A resident is defined in a different class than a citizen in terms of the rights to which he or she is entitled. In Israel residents are covered by the National Health Insurance. Residency status was granted to Arab residents of Jerusalem after the 1967 war.

There were no significant differences found between Jews and Arabs regarding age and gender. Significant differences were found regarding marital status and the number of children: more Arab patients were married and the median number of children was higher, as compared to Jewish patients.

In terms of health status, there were significant differences between Jews and Arabs. Jewish patients reported better health statuses compared to Arab patients. They had fewer chronic illnesses, required fewer medications on a regular basis, and reported less chronic pain.

In terms of education and socioeconomic status, there were significant differences between the study groups, with lower levels on both variables in the Arab group compared to those of the Jewish group. No significant difference was found in employment rates. The percentage of Arab patients who did not speak Hebrew (nearly half) was significantly higher than the percentage of Jewish patients who did not speak Hebrew. Most of the Arab participants in this study held the status of permanent resident in Israel, distinct from the status of citizen.

Significant differences were found in terms of feeling safe and having social support. A greater proportion of Arab than Jewish patients used welfare services, and a greater proportion likewise reported having experienced physical and mental violence, expressed concern about their safety, and felt they had fewer social support resources. Regarding illegal drug use, a significant difference was found, whereby a greater proportion of Jewish patients reported having used drugs sometime in the past or present. Regarding alcohol use, not enough information was reported.

There were significant differences in some of the characteristics of health service utilization. A greater proportion of Arabs than Jews reported encountering language barriers when seeking healthcare in the community. A higher percentage of Arab participants did not seek information about medical rights, compared to the Jewish patients, who reported seeking information but encountered difficulties. A small percentage of Arab participants reported that their family physicians were not fluent in Hebrew, meaning they were unable to read hospital discharge letters written in Hebrew.

No significant differences were found between the groups in the percentage of people who gave up community healthcare services because of financial constraints (5.3% among Arabs, and 5.5% among Jews). There was also no significant difference between the groups for those who did not have a designated family physician in the community (7% of Arabs and 12% of Jews do not have a designated family physician).

### Emergency department utilization patterns and current ED visit by ethnicity

The characteristics of ED utilization patterns in the two study groups are shown in Table 2.

**Table 2:** Characteristics of ED utilization patterns and current ED visit

**Characteristic**		**Arabs (*n* = 170)**	**Jews** **(*n* = 257)**	***p* value**
Previous ED visits last year, %	0	60.4	56.3	U = 20,468 *P* = 0.440
	1–5	32.5	36.5	
	6–10	5.9	4.0	
	More than 10	1.2	3.2	
Repeat visits within 30 days, %		16.9	9.3	x^2^ = 5.225 *p* = 0.022
Mode of arrival to ED, %	Ambulance (compared with self-arrival by a car or public transport)	36.7	31.1	x^2^ = 1.433 *P* = 0.231
Duration of Arrival, %	More than an hour	5.9	5.5	x^2^ = 0.028 *P* = 0.868
Professional referral, %	No professional referral	35.5	26.9	x^2^ = 1.650 *P* = 0.199
Previous medical application, %		57.5	46.9	x^2^ = 4.535 *P* = 0.033
Patient escorts, %	Have any accompanies	82.2	67.2	x^2^ = 11.764 *P* = 0.001
Translator (for those who had to use a translator), %	Escort	58.1	54.5	x^2^ = 3.357 *P* = 0.187
	Professional translator	1.1	9.1	
	Non-professional translator	40.9	36.4	
ED patient experience, Median (IQR)^a^		4.00 (3.75–4.50)	4.25 (3.50–5.00)	U = 18,280 *p* = 0.030
Feeling of discrimination, %		1.6	4.7	x^2^ = 3.702 *p* = 0.054

^**a**^ Average of the four questions related to the ED experience. Higher score means more positive experience

The characteristics of the current visit to the ED were similar between the two groups in terms of how they arrived at the ED and the time lapse between the emergency and the arrival. Most patients arrived independently (not by an ambulance); there were no reports of delays at military checkpoints, and in both groups, only a small minority reported that it took them more than one hour to arrive. Also, there was no difference as to who referred the patient to the ED. Most respondents came to the ED after consulting with a professional (physician, paramedic, medic, or other medical staff). However, there was a significant difference between the groups regarding prior medical consultation. A higher percentage of Arab patients than Jewish patients had visited another medical center (such as an HMO clinic or a community urgent care clinic) before arriving at the ED. There was no difference between the groups’ reports regarding experiencing discrimination in the ED. There was a significant difference between the groups in terms of their patient experience in the ED, with a higher median satisfaction found in the Jewish group.

In terms of the frequency of ED visits over the last year, there was no difference between Jewish and Arab patients. However, when comparing repeat visits over the previous month, there was a significant difference: a higher rate of Arab patients than Jewish patients returned to the medical center over the previous month for the same reason (16.9% vs. 9.3%).

### Univariate analysis of repeat visits

Given the multiple differences found between the Jewish and Arab patients regarding the characteristics of the ED visits, we conducted a separate analysis for each group, to determine the relationships between the patient characteristics and the dependent variable: repeat ED visits within one month for the same reason. The findings are shown in Table 3.

**Table 3:** Repeat visits ^**a**^ by sociodemographic characteristics, for Jews and Arabs

		**Jews** **% of those with repeat visits in each category**		**Arabs** **% of those with repeat visits in each category**	
		Total n for repeat visits = 23		Total n for repeat visits = 27	
Age	mean (SD)	yes	no	yes	no
		55.5 (21.1)	51.59 (23.6)	53.59 (16.5)	50.73 (18.9)
	***p* value**	**0.457**		**0.466**	
Gender	Male	8.7		18.6	
	Female	9.9		14.9	
	***p* value**	**0.733**		**0.529**	
Marital status	Single	7.4		11.1	
	Married	8.8		17.8	
	Divorced / separate / widower	13.0		16.7	
	***p* value**	**0.598**		**0.776**	
Health status	Very good	7.0		9.8	
	Fair / good	7.6		19.0	
	Not very good	12.9		20.5	
	Poor	50.0		25.0	
	***p* value**	**0.004**		**0.113**	
Chronic disease	No chronic disease	7.6		10.0	
	One or more chronic disease	11.0		20.4	
	***p* value**	**0.357**		**0.107**	
Chronic medicine	No chronic medicines	5.0		11.1	
	Chronic medicines	13.9		19.8	
	***p* value**	**0.017**		**0.165**	
Chronic pain	No chronic pain	7.4		18.8	
	Sometimes	17.4		14.3	
	More than a month	10.0		12.5	
	More than 6 months	10.8		16.7	
	***p* value**	**0.393**		**0.748**	
Social support	Social support	8.1		22.7	
	Deficient social support	20.0		14.7	
	***p* value**	**0.772**		**0.223**	
Welfare services	Not familiar	9.4		12.8	
	Familiar	8.6		26.5	
	***p* value**	**0.877**		**0.035**	
Concern about personal safety	Seldom / never	8.7		13.7	
	Always / sometimes	13.2		27.8	
	***p* value**	**0.386**		**0.047**	
Education	No formal education / elementary school	4.3		18.9	
	Secondary school	14.8		18.4	
	Higher education	7.0		8.3	
	Religious seminary (Yeshiva)	7.7		–	
	***p* value**	**0.223**		**0.334**	
Employment	Not employed	13.0		21.6	
	Employed	5.3		8.8	
	***p* value**	**0.039**		**0.039**	
Socioeconomic status	Poor	23.8		24.0	
	Not so good	12.1		11.8	
	Fair / good	8.8		17.5	
	Excellent	6.8		19.0	
	***p* value**	**0.032**		**0.908**	

^**a**^ Repeat ED visits are defined as a return visit (more than one) within one month for the same reason

The results revealed similar trends in the two groups in terms of the following patient characteristics: employment, health status, concern for personal safety, frequency of visits to the PCP, and foregoing community healthcare services due to financial constraints.

In both groups, unemployed participants were more likely to visit the ED more than once in the last month. The worse the self-reported health status was, the more likely the patients were to return to the ED. However, in the Arab group, this relationship was not statistically significant. The trend was also similar among chronic medication users, who were more likely to return to the ED than were their counterparts. Mostly among Arab patients, concern for personal safety was positively associated with repeat visits. In the Jewish group, the trend was similar, but the association was not statistically significant.

Frequent PCP visits (more than 10 times a year) in both groups were related to repeat visits to the ED. Deferring healthcare services because of financial constraints was also associated with a high rate of repeat ED visits in both groups, but the association was not significant in either.

Regarding other variables, we found different trends in the two groups. The use of welfare services was related to repeat visits only in the Arab group. In the Jewish group, a lower socioeconomic level was significantly related to repeat visits to the ED. In the Arab group, by contrast, the direction of the association was not consistent.

No other variable was associated with repeat ED visits. This includes the variables of age, gender, and marital status.

### Multivariate analysis

We chose to include in the multivariate models the variables that in the univariate analysis were found to be significantly associated with the dependent variable. Before introducing the variables in the multivariable model, we examined collinearity to verify that there were no strong associations between the variables (*r* > 0.7).

Among the Jewish patients, health and socioeconomic status were linked to repeat visits to the ED. More specifically, poor health status was associated with a greater number of repeat visits. Poor economic status was positively linked to repeat visits to the ED. Among the Arab patients, repeat visits to the ED were related to concerns about personal safety.

## Discussion

This study examines social and structural factors and their relationship to patterns of the use of health services in an ED in Jerusalem that serves a mixed population. We found that the pattern of repeat visits to the ED for the same reason within a month in both ethnic groups was associated (Arabs and Jews) with health status and frequent visits to a family physician. Among the Arab patients, this pattern was associated also with concerns about personal safety, whereas among the Jewish patients, it was associated with poverty.

### Sociodemographic differences

The study found significant differences in sociodemographic characteristics of the Jewish and Arab ED visitors, including differences in their economic status, education levels, and employment rates. Also, differences were related to the degree of knowledge about national welfare services, concerns for personal safety, and the availability of social support. These data reflect the existing gaps between Jews and Arabs in Israel, in general, and in Jerusalem in particular [24].

Consistent with other studies [5, 23], we found that the reported health status was worse among Arab ED patients, in terms of overall health condition, the percentage of patients on chronic medication, and the degree of chronic pain. Almost half of the Arab ED patients had more than one chronic disease. The correlation between socioeconomic status and health status was in line with the findings of the studies presented in the literature review [15, 30]. This finding illustrates the structural vulnerability of the Arab population in Israel. In other words, inequalities between groups in society have a detrimental effect on their health status [30].

### ED repeat visits

Considering the relationship demonstrated in previous studies between structural vulnerability and ED repeat visits [16, 17], we sought to examine the relationship between ethnicity and ED utilization patterns, and the association between these patterns and social and structural factors. The ED utilization pattern on which we focused in this study was the overuse of healthcare services as reflected in repeat ED visits. The “repeat ED visits” variable was defined as returning to the ED within a month of the previous visit for the same reason.

There was a difference between Jews and Arabs in terms of repeat visits, with significantly more recurring visits among the Arab patient group (16.9% vs. 9.3%). In the hospital where the study was conducted, the rate of general repeat visits as of 2017 was 14.1% [31], indicating that the rate of repeat ED visits of the Arab participants was higher than the overall rate of general repeat visits to the hospital. These findings support those of previous studies, which have shown that disadvantaged populations are more likely to use ED services [13, 14].

Among the Jewish patient group, when controlling for other variables, repeat ED visits were associated with perceived poor health, poverty, and frequent visits to a family physician. These findings are consistent with findings from previous studies that examined factors associated with frequent ED inquiries [32, 33]. As was found in previous studies, not surprisingly, poor health is significantly associated with repeat ED visits [18, 34, 35]. However, among the Arab participants in the current study, perceived bad health status was not significantly related to repeat ED visits.

Poor economic status and lack of employment have been associated in previous studies with repeat ED visits of various populations [36–39]. The current study found a similar relationship, as those who were poor were more likely to return to the ED.

In Israel, there is a national health insurance law that provides medical insurance to all Israeli residents, including the Arab residents of East Jerusalem (National Health Insurance Law, 1994). It seems that despite the existence of insurance, the financial situation still influences repeat ED visits. The claim that lack of health insurance influences health status is supported by several studies [16], but not by others [40]. Soril et al. compared different healthcare systems and found that repeat visitors to the ED shared similar characteristics [35]. They found that low economic status was related to healthcare overuse (as in repeat ED visits) in both privatized health systems (e.g., in the United States) and in national health insurance systems.

The differences in repeat ED visits can reflect healthcare inequalities resulting from poverty. In 2013, the Israeli Committee for the War Against Poverty (called the *Elalouf* Commission) stated the following: “The government and its institutions must recognize social (poverty) determinants of health and declare reduction of health inequality as a strategic goal.” The commission’s recommendations included, among other things, opening information centers to enable healthcare users to exercise their rights as well as providing discounts on medications for treating chronic conditions of patients who live in poverty. Another recommendation was to provide healthcare workers with comprehensive information on the association between poverty and health [41]. The findings of the present study help to explain and substantiate the association between poverty and health status and thus may further promote awareness of this issue among healthcare students and workers.

The findings indicate that most of the variables that were found in both univariate and multivariate models to be significantly related to repeat ED visits among the Jewish patients were found to be significant—or at least in the same trend—among the Arab patients as well. However, in contrast with the finding pertaining to the Jewish patients, economic status was not directly related to the pattern of ED use among the Arab patients. People of poor financial status and people of excellent financial status both reported the greatest number of repeat visits, whereas people who defined their financial status as less than excellent, but reasonable or better than poor, reported fewer recurring ED visits.

Findings from previous studies have shown that repeated ED visits are inversely related to the availability, accessibility, and quality of community health services. A study conducted in Israel indicated that among the Arab population, the availability of healthcare services in the community (physical availability, accessibility, as well as the ability and tendency to utilize them) was much lower than among the Jewish population [23]. In addition, according to previously published surveys, poverty and economic hardship affect access to health services [42].

It is possible that among the population of ED visitors who participated in this study, the Jewish participants had greater access than did their Arab counterparts to good quality healthcare services. Such services are less available and less accessible to people from disadvantaged socioeconomic levels, who consequently resort to repeat ED visits. Accordingly, for Arab individuals living in disadvantaged neighborhoods or in remote villages, access to community health services medical services is limited regardless of the socioeconomic level, which might explain the lack of association between the economic status and frequent ED visits.

Arab residents of East Jerusalem are an example of a population with less availability and poorer quality of healthcare services, which are often provided in their residential areas by external suppliers and not directly by main Israeli HMOs as in all other areas of the country. This initiative to use external suppliers (outsourcing) was approved by the Ministry of Health: “Considering the constraints…. [it is] an unavoidable necessity, which provides those affiliated with the various HMOs reasonable access to services.” [43]. That is, although the Ministry of Health recognized the disadvantages of this approach, it was considered a necessary step. However, it is unclear why despite this imperfect solution, both wealthier and disadvantaged Arab residents of East Jerusalem have more repeat visits, relative to their neighbors who defined their socioeconomic status as middle class.

Chernichovsky et al., 2017 [23] discussed patterns of use of healthcare services among the Arab citizens of Israel. They found that they tended to demonstrate relatively ineffective usage patterns, meaning fewer visits to health specialists, more visits to family physicians, and more hospitalizations. These findings were confirmed by the results of the present study, which indicated a significant difference between the two groups in the number of visits to a family doctor, with a higher number of visits reported by Arab participants. However, in both groups, repeat ED visits were associated with frequent visits to a family physician. This finding is supported by studies conducted outside of Israel, which demonstrated that frequent use of healthcare services also included frequent visits to the family physician [13, 34]. This pattern suggests that repeat visits to a family physician do not necessarily preclude visits to the hospital. In Israel, the positive association may be even stronger, because obtaining a medical referral to the ED beforehand covers all the costs of the hospital visit. According to a survey by the Brookdale Institute in 2019, about one-third of appointments with family physicians in all HMOs are scheduled for the single purpose of obtaining referrals and filling out health-related forms [42].

Among the Arab participants in the current study, the only variable associated with repeat visits, when controlling for other variables, was a concern for personal safety. Using this measure revealed a significant difference between the two groups, with the Arab participants expressing greater concern about their safety than did their Jewish counterparts.

The relationship between referral patterns and concern for personal safety has been examined following the discussion in the US regarding the misuse of healthcare services and its association with lack of safety reported by vulnerable groups [15, 44]. An earlier study had found an association between health status and a sense of personal insecurity [45]. These findings may reflect the relationship found in other studies between exposure to violence and repeat visits [46, 47]. In this study, no association was found. It is possible that actual personal exposure to violence in this study was underreported. It is possible that the sense of personal insecurity, reflecting many facets of life—economic, social, and physical—also affects the pattern of repeated visits.

The findings of this study help to bridge the gap of knowledge regarding factors associated with repeated ED visits among Arab and Jewish patients in Jerusalem. The study contributes to the understanding of the association between social and structural factors and the use of health services and strengthen the knowledge about structural inequalities and structural vulnerability of minority populations.

Buchbinder linked repeat ED visits with low socioeconomic status, explaining that staff structural competency plays an important role in preventing repeat ED visits associated with poverty-related reasons [48]. When the team recognizes and understands the factors related to referral patterns, they should refer the patient to appropriate resources that can address the problem that led to the unnecessary ED visit. Thus, the medical team in the ED can recognize and respond to structural inequalities and act as a gatekeeper, capable of referring and redirecting patients to effective health resources. A team that has been properly trained to do this will exhibit a sensitive understanding of the local health system and its potential barriers. Understanding the factors that can influence recurring visits, such as ethnicity and economic hardship, will enable structurally qualified caregivers to provide effective and appropriate care.

### Limitations

Alongside the advantages of the study which seeks to examine the structural determinants in the unique context of emergency medicine in Jerusalem, there are some limitations. First, the data for this study were gathered using a structured questionnaire, parts of which had not been used previously and therefore had not been validated before. The questionnaire was constructed based on previous qualitative exploratory research. Following a pilot survey of several ED visitors—Jews and Arabs—the questionnaire was examined by experts and a reassessment of the research tool was made. However, this is the first study in which this questionnaire was used.

In the data analysis, we included both study populations, Arabs and Jews, as separate groups. It is important to note that each one of the groups consists of diverse subgroups with unique characteristics. The analysis presented in the study did not take those into account. For example, utilization patterns among the ultraorthodox may differ from the rest of the Jewish population. Similarly, there may be differences between Christian and Muslim Arabs. These differences between the subgroups may affect the associations.

Since the study was conducted in only one hospital in Jerusalem, its findings may represent only the caregivers and patients of this hospital, and might not be valid for other hospitals serving different populations. However, as discussed above, most of the findings that emerged in the present study were consistent with those in the established literature.

In this study, a convenience sample was used. However, there was an attempt to sample participants in all shifts to reduce selection bias. Data were collected from patients who were already in the hospital’s ED. Hence, information about the chain of events that preceded the participants’ arrival at the ED could not be sufficiently examined. This study did not find any differences in means of access to the ED; however, people who typically encounter barriers to emergency medical services might not have had access to this ED and were not present at the time of the study, so no information could be obtained. This absence may pertain to people without medical insurance or those who live beyond the military checkpoints. In addition, the study did not examine the outcomes of repeat visits for the patients.

## Conclusion

This study found associations between social and structural factors, and the ED referral pattern. The pattern of repeated visits to the ED for the same reason within a month was associated in both ethnic groups with health status and frequent visits to a family physician. Among the Arab patients, repeated visits to the ED were associated also with concerns about personal safety, whereas among the Jewish patients, it was associated with poverty. In addition, many differences between Jewish and Arab patients were found in terms of both sociodemographic and socioeconomic levels, as well as in the pattern of ED use. These findings illustrate the existing gaps and the vulnerability of disadvantaged populations, which affects their use of healthcare services.

Knowledge and understanding of these issues on the part of the healthcare team, in tandem with solutions formulated at the institutional and political levels, can promote equity by providing structurally competent care. Promoting the recognition of structural forces is the first step in developing better understanding, discourse, and practice in the clinical setting [49–51]. This study sheds light on the unique needs of a variety of patients in an ED in Jerusalem, on the social and structural factors related to their access to care in EDs, and on the necessity to train the healthcare staff to better meet the various patients’ needs. Identifying and understanding the social and structural factors that influence the quality of care helps develop healthcare providers’ competence and improve healthcare policies [27].

Key recommendations drawn from the study include the need to formulate a policy that will ensure that disadvantaged populations have better access to medical specialists and their services in the community; the need to strengthen these populations’ knowledge of, access to, and understanding of their medical rights; and the need to use the translation services provided by the Ministry of Health and the hospitals after adapting them to the particular demands of the ED workflow. In future studies, it would be interesting to examine these issues and conduct comparisons with other minority groups, such as the ultraorthodox Jewish community, elderly, or people with disabilities, who were not addressed in this study.

## Supplementary Information

**Additional file 1. **Questionnaire items.

## Data Availability

The datasets used and/or analyzed during the current study are available from the corresponding author upon reasonable request.

## Abbreviations

ED: Emergency Department
HMO: Health Maintenance Organization
IQR: Interquartile Range
OR: Odds Ratio
PCP: Primary Care Physician
