# Supplementary material for: Social and structural determinants of emergency department use among Arab and Jewish patients in Jerusalem
Source: Int J Equity Health. 2022 Nov 7;21:156. doi: 10.1186/s12939-022-01698-1 (PMC9641903; doi:10.1186/s12939-022-01698-1)
Supplement: Supplementary file 1 — Additional file 1. Questionnaire items. [file 12939_2022_1698_MOESM1_ESM.docx]

**Additional file 1 – Questionnaire items**

| **sociodemographic details** |
| --- |
| age, gender, city, neighborhood, country of birth, religious group, origin, marital status, number of children, citizenship status, education, employment |
| **health status** |
| How do you usually assess your health? (very good, reasonable, not so good, poor) |
| Do you have an illness or health condition that you suffer from and / or require regular medication or other medical treatment? |
| Do you take medication regularly? |
| Do you suffer from pain regularly? (> half a year, > 1 month, occasionally, no) |
| **current ED visit** |
| How did you get to the emergency room today? |
| How long did it take you to get to the emergency room from the moment you left? |
| Did you cross a checkpoint (security / army) on the way? |
| Who advised you to go to the emergency room? |
| Did you have the option to choose the emergency room where you wanted to be treated? |
| Who was involved in the decision to go to this hospital? |
| Why did you come to the ED today? |
| Before your emergency room visit, have you visited another medical center (today)? |
| Did you come accompanied by relatives or friends? |
| What languages ​​do you speak? |
| What language is most comfortable for you to speak? |
| Did you use a translator in the ED? |
| If so, who was the translator? |
| Where do you plan to go after discharge? |
| What is your general satisfaction with the stay in the emergency room (until now) from 1 (not satisfied at all) to 5 (very satisfied)? |
| How satisfied are you with the treatment given to you so far from 1 (not satisfied at all) to 5 (very satisfied)? |
| To what extent are the explanations you receive clear and understandable to you? |
| To what extent do you feel you are being treated with good hands? |
| To what extent do you feel you are being treated with good hands? |
| **Health services utilization** |
| Are you insured with any health insurance? |
| Does your family doctor speak Hebrew (Can he understand hospital discharge letters written in Hebrew)? |
| How many times have you visited your PCP in the last year? (0, 1-5, >5, >10) |
| How many times have you been in ED (any ED) in the last year? (0, 1-5, >5, >10) |
| When was your last visit to the ED (the time before the current visit)? |
| Did you go to the emergency room in the past for the reason you came here now? |
| Do you have difficulty finding information about patient rights in the healthcare system? |
| Have you ever used informal health services? |
| Have you ever given up on medical care that you needed / that was recommended to you / that you were referred to it? |
| Have you ever given up on purchasing medications that you needed / that your doctor prescribed for you? |
| Have you had difficulty getting health care because of language problems (in the community)? |
| **Socioeconomic background** |
| Do you feel you have support from friends or family when you need it? (always, usually, sometimes, never) |
| Have you used welfare services in the past? |
| Have you experienced physical violence in the past year? |
| Have you experienced any other violence in the last year more than you can bear? |
| Do you ever worry about your personal safety? |
| Which of the following is true about you regarding drug use (including medical cannabis use)? (take regularly, take occasionally, rarely take, medical cannabis, have taken in the past) |
| Which of the following is true about you regarding drinking alcohol? (several times a day, once a day, several times a week, < once a week, never) |
| The median salary in Israel is 7,452 NIS per month. Compared to this figure, how do you define your financial situation? |
| How do you define your financial situation in relation to the people in your immediate environment? |
